# Metabolic profiling of pre-gestational and gestational diabetes mellitus identifies novel predictors of pre-term delivery

**DOI:** 10.1186/s12967-020-02531-5

**Published:** 2020-09-24

**Authors:** Ilhame Diboun, Manjunath Ramanjaneya, Yasser Majeed, Lina Ahmed, Mohammed Bashir, Alexandra E. Butler, Abdul Badi Abou-Samra, Stephen L. Atkin, Nayef A. Mazloum, Mohamed A. Elrayess

**Affiliations:** 1 Hamad Bin Khalifa University (HBKU), Doha, Qatar; 2 Qatar Metabolic Institute, Hamad Medical Corporation, Doha, Qatar; 3 Translational Research Institute, Hamad Medical Corporation, Doha, Qatar; 4 Weill Cornell Medicine-Qatar, Doha, Qatar; 5 Diabetes Research Center (DRC), Qatar Biomedical Research Institute (QBRI), Hamad Bin Khalifa University (HBKU), Qatar Foundation (QF), PO Box 34110, Doha, Qatar; 6 Royal College of Surgeons in Ireland Bahrain, Adliya, Kingdom of Bahrain; 7 Biomedical Research Center (BRC), Qatar University, Doha, Qatar

**Keywords:** Metabolomics, Pregnancy, Gestational diabetes, Type 2 diabetes, Pre-term delivery

## Abstract

**Background:**

Pregnant women with gestational diabetes mellitus (GDM) or type 2 diabetes mellitus (T2DM) are at increased risks of pre-term labor, hypertension and preeclampsia. In this study, metabolic profiling of blood samples collected from GDM, T2DM and control pregnant women was undertaken to identify potential diagnostic biomarkers in GDM/T2DM and compared to pregnancy outcome.

**Methods:**

Sixty-seven pregnant women (21 controls, 32 GDM, 14 T2DM) in their second trimester underwent targeted metabolomics of plasma samples using tandem mass spectrometry with the Biocrates MxP^®^ Quant 500 Kit. Linear regression models were used to identify the metabolic signature of GDM and T2DM, followed by generalized linear model (GLMNET) and Receiver Operating Characteristic (ROC) analysis to determine best predictors of GDM, T2DM and pre-term labor.

**Results:**

The gestational age at delivery was 2 weeks earlier in T2DM compared to GDM and controls and correlated negatively with maternal HbA1C and systolic blood pressure and positively with serum albumin. Linear regression models revealed elevated glutamate and branched chain amino acids in GDM + T2DM group compared to controls. Regression models also revealed association of lower levels of triacylglycerols and diacylglycerols containing oleic and linoleic fatty acids with pre-term delivery. A generalized linear model ROC analyses revealed that that glutamate is the best predictors of GDM compared to controls (area under curve; AUC = 0.81). The model also revealed that phosphatidylcholine diacyl C40:2, arachidonic acid, glycochenodeoxycholic acid, and phosphatidylcholine acyl-alkyl C34:3 are the best predictors of GDM + T2DM compared to controls (AUC = 0.90). The model also revealed that the triacylglycerols C17:2/36:4 and C18:1/34:1 are the best predictors of pre-term delivery (≤ 37 weeks) (AUC = 0.84).

**Conclusions:**

This study highlights the metabolite alterations in women in their second trimester with diabetes mellitus and identifies predictive indicators of pre-term delivery. Future studies to confirm these associations in other cohorts and investigate their functional relevance and potential utilization for targeted therapies are warranted.

## Background

Gestational diabetes mellitus (GDM) represents any degree of glucose intolerance with onset during pregnancy, regardless of whether treated by insulin or diet modification, or whether the condition persists after pregnancy or not [1]. It does not exclude the possibility that unrecognized glucose intolerance may have antedated or begun concomitantly with the pregnancy. GDM represents one of the most frequent complications in pregnancy [2]  with a prevalence range of 1–14% based on the diagnostic criteria, study population, ethnicity and geographical location [3]. Its increasing prevalence has been attributed to the obesity epidemic among women of reproductive age [4]. GDM is diagnosed when glucose levels are elevated in the late second trimester [5]. Postpartum, 20% of women with GDM develop impaired fasting glucose (IFG) and/or impaired glucose tolerance (IGT), causing a 7.4 times higher risk of type 2 diabetes mellitus (T2DM) later in life compared to matching controls [6] and have an increased risk of cardiovascular disease (CVD) [7]. GDM has been associated with adverse outcomes including pre-term delivery, preeclampsia, macrosomia, perinatal mortality and neonatal metabolic complications [8]. Spontaneous pre-term delivery has been linked to poor glycemic control and parity [9–12], and is associated with a higher risk for neonatal intensive care unit admission due to respiratory failure and hypoglycemia [13]. It is also associated with chronic respiratory disease, ischemic heart disease and metabolic disorders [14–16]. These adverse outcomes of GDM have led clinicians to implement various strategies including fetal surveillance and induction of labor [17, 18]. A number of risk factors for GDM have already been identified, including maternal age, family history of diabetes, pre-pregnancy obesity, and multiple pregnancies [19]. However, the metabolic pathways in GDM and/or pre-gestational T2DM in pregnancy and their relationship to pregnancy outcomes is poorly understood. The identification of novel biomarkers may, therefore, have clinical diagnostic and therapeutic applications.

Discovery of metabolic mediators underlying disease progression in obesity-associated insulin resistance and T2DM [20] were facilitated by advancing metabolomic tools such as mass spectrometry (MS) technologies, providing a better understanding of the etiology of the disease. The metabolic signature differentiating healthy controls from individuals at higher risk of T2DM included various carbohydrates (e.g. glucose and fructose), lipids (e.g. phospholipids, sphingomyelins, and triglycerides), and amino acids (branched-chain amino acids, aromatic amino acids, glycine, and glutamate) [20–22]. The pathophysiological changes that occur in pre-gestational T2DM and GDM are similar [23]. However, metabolomic studies aimed at predicting risk of GDM in pregnant women have shown inconsistent findings, as some reported lower blood creatinine, trimethylamine-N-oxide, and betaine, while others reported elevated acetylcarnitines, bile acids, ketones, creatinine, carbohydrate, and other lipids and organic acids [24]. However, few studies have investigated the association between these metabolic markers and risk of pre-gestational T2DM and GDM-associated pathologies including pre-term labor [25].

The aim of this study was to perform targeted metabolomics analysis of blood samples from pregnant women in their second trimester with pre-gestational T2DM, GDM or matching healthy controls to investigate the metabolic pathways underlying these pathologies and identify potential predictors of increased risk of pre-term labor.

## Methods

### Study design

This was a cross sectional study in 67 pregnant women (21 controls, 32 GDM, 14 T2DM) who were recruited during their second trimester at the antenatal clinic at The Women Wellness and Research Center of Hamad Medical Corporation (GDM and pre-gestational T2DM) in Doha, Qatar. Protocols were approved by Institutional Review Boards (IRBs) of the Hamad Medical Corporation (15101/15) and Weill Cornell Medical College in Qatar (15-00016). Demographics, anthropometrics and medical history data were collected including age, ethnicity, socio-economic background, vital signs, height, weight, menstrual cycle, period of infertility, medications, complications, comorbidities and family medical history. All pregnant women are screened in the first antenatal care visit using fasting blood glucose (FBG). If the FBG at the first visit is < 5.1 mmol/l (92 mg/dl); 75 g oral glucose tolerance test (OGTT) is performed at 24 weeks’ gestation. The world health organization (WHO) criteria [FBG ≥ 5.1 mmol/L (92 mg/dl), 1 h post OGTT ≥ 10.0 mmol/L (180 mg/dl) or 2 h post OGTT ≥ 8.5 mmol/L(153 mg/dl)] is used to diagnose GDM. GDM patients were started on diet for 2 weeks with the aim of a FBG ≤ 5.3 mmol/l (95 mg/dl) and the 2 h post prandial glucose being ≤ 6.8 mmol/l (120 mg/dl) in ≥ 80% of the readings. If more than 20% of the readings were above target then Metformin therapy was implemented and increased incrementally followed by insulin supplementation when glucose targets were not achieved. Women with Type 2 diabetes were all treated with Metformin and basal-bolus insulin.

Laboratory tests included second trimester full blood count, biochemical profile and thyroid function tests. Blood samples were collected for the metabolomics analysis. All patients gave their written informed consent and the conduct of the study was in accordance with the International Council for Harmonisation Good Clinical Practice and the Declaration of Helsinki. Pregnancy outcomes of gestational age at delivery, birthweight, maternal weight, blood pressure and foetal outcome were recorded and collated with the metabolomic profile for all subjects who participated in the study.

### Metabolomics

Targeted metabolomics of plasma samples was performed using tandem mass spectrometry with the Biocrates MxP^®^ Quant 500 Kit (Biocrates, Innsbruck, Austria) at the Fraunhofer Institute for Toxicology and Experimental Medicine (ITEM). Lipids were measured by Flow Injection Analysis Tandem Mass Spectrometry (FIA-MS/MS) using a 5500 QTRAP^®^ instrument (AB Sciex, Darmstadt, Germany) with an Electrospray ionization (ESI) source, and small molecules were measured by Liquid chromatography–mass spectrometry (LC–MS/MS) using the same 5500 QTRAP^®^ instrument as previously described [26]. Briefly, a 96-well based sample preparation device was used to quantitatively analyze the metabolite profile in the samples. This device consists of inserts that have been spotted with internal standards, and a predefined sample amount was added to the inserts. Next, a phenylisothiocyanate solution was added to derivatize some of the analytes (e.g. amino acids), and after the derivatization was completed, the target analytes were extracted with an organic solvent, followed by a dilution step. The obtained extracts were then analyzed by FIA-MS/MS and LC–MS/MS methods using multiple reaction monitoring (MRM) to detect the analytes. Data were quantified using appropriate MS software (Sciex Analyst^®^) and imported into Biocrates MetIDQ™ software for calculating analyte concentrations, data assessment and compilation.

### Statistical analysis

Demographics traits analysis: Statistical analyses were carried out using IBM SPSS version 25, R version 3.2.1 and SIMCA 14 software (Umetrics, Sweden). Variables with skewed distributions were log transformed to ensure normality [27]. Comparisons were performed with t-test, Wilcoxon–Mann–Whitney and 1-way ANOVA as appropriate. Significance was defined as P ≤ 0.05. Non-parametric tests were used for comparing ordinal or non-normal variables. Metabolomics data analysis: Principle component analysis (PCA) was performed using R version 2.14, www.r-project.org/. PCA revealed two main components (PC1 and PC2) that together captured 24% of the variance in the data. Orthogonal partial least square discriminant analysis (OPLS-DA), implemented as part of the software SIMCA, was used to compare controls, GDM and T2DM groups. OPLS-DA is recommended in cases of regression where the number of explanatory variables is high, and where it is likely that the explanatory variables are correlated as it is the case in our data. All metabolites with a percentage of missing values greater than 50% were excluded from SIMCA analysis. Linear regression was performed to identify significant metabolites differentiating study groups (controls vs GDM and T2DM) and (Controls vs GDM + T2DM) using the R statistical package (version 2.14, www.r-project.org/) after correcting for age, BMI and principle components (PC1 and PC2). Additionally, the gender interaction effect was evaluated in ANOVA model that featured the same confounders. Contrast analysis was conducted using R package Emmeans to pinpoint the significance of effect per gender group. Function enrichment analysis was performed using Fisher’s exact test by considering metabolites with a nominal p-value less than 0.1 from linear regression analysis. For a given biological function, the test assesses the probability of observing the associated nominally-significant metabolites from the linear model by pure chance. The biological categories tested for enrichment were provided by Biocrates and expanded manually by reference to the Human Metabolome Database [28]. The Elastic net regularization of linear models, implemented in R package GLMNET, was used for selection of best predictors of clinical traits of interest to this study.

## Results

### General characteristics of participants

**Fig. 1:**
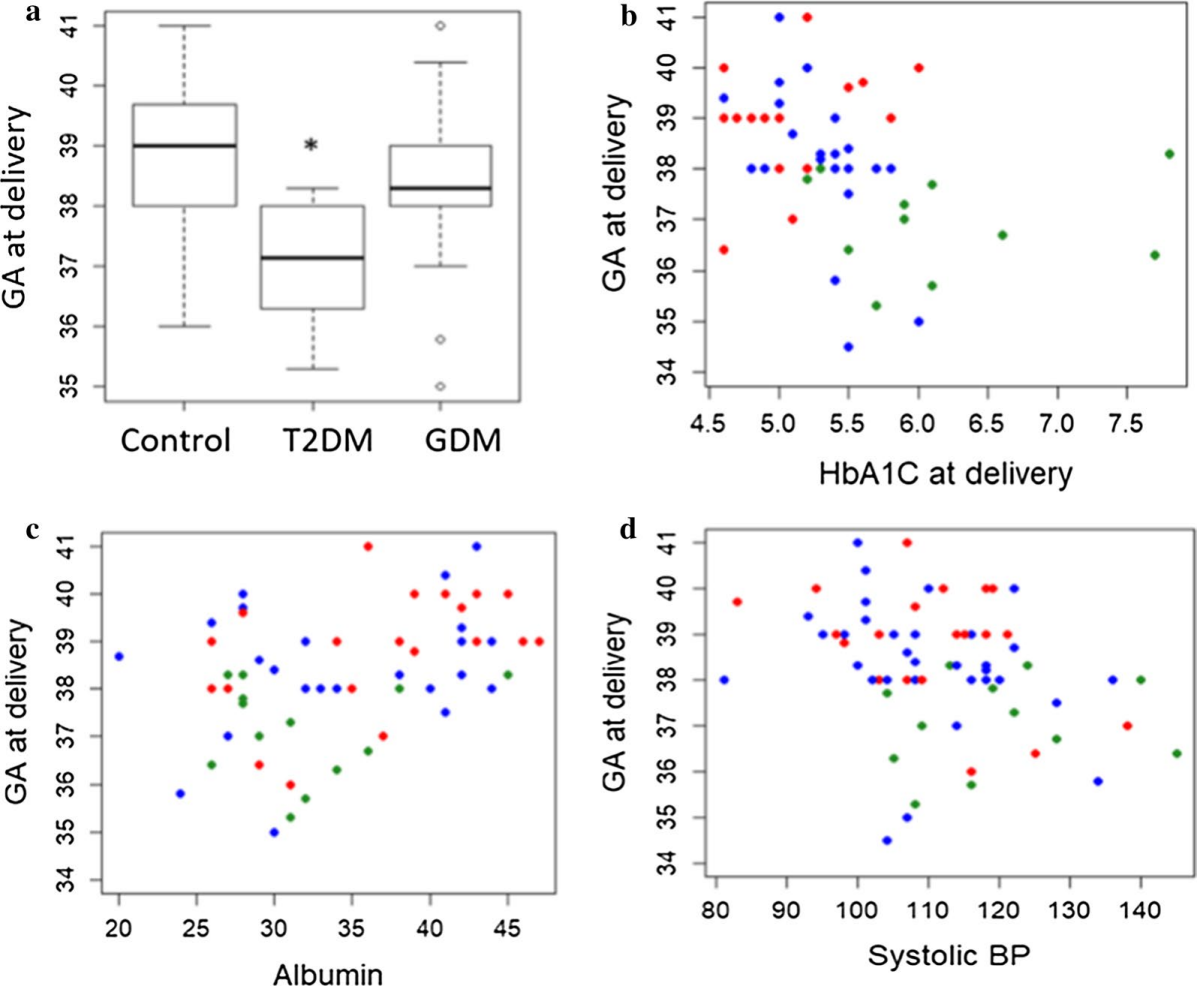
Differences in gestational age (GA) at delivery in controls, gestational diabetes (GDM) and type 2 diabetes (T2DM) groups (**a**). Correlation of gestational age at delivery with baseline HbA1c (**b**) systolic blood pressure (**c**) and serum albumin (**d**). GA at delivery was compared among controls, GDM and T2DM groups using ANOVA. Pearson’s correlation was used to assess the relationship between GA at delivery and serum HbA1c, albumin and systolic blood pressure measured at third trimester. p-value significance level of 0.05 (*) was used

67 young (31.9 ± 5.7 years) overweight/obese (31.9 ± 6.9 kg/m^2^) pregnant women were included in this study. All included pregnancies resulted in single births. The T2DM women were older, had higher triacylglycerols (TG), insulin and glycated haemoglobin (HbA1c), than body mass index (BMI)-matched controls and GDM (p < 0.05) (Table 1). Gestational age (GA) at delivery was on average 2 weeks earlier in T2DM than controls and GDM (p = 0.01) (Fig. 1a). GA at delivery was negatively correlated with HbA1c at baseline (R = − 0.34, p = 0.01, Fig. 1b) and with systolic blood pressure (SBP) at baseline (R = − 0.34, p = 0.005, Fig. 1c), but positively correlated with serum albumin (R = 0.39, p = 0.002, Fig. 1d). A regression model, incorporating explanatory variables (study group, HbA1c, SBP and serum albumin), indicated independent significant association of all four covariates with GA at delivery (study group: Beta = − 1.7, HbA1c: Beta: − 0.85, p = 0.01, SBP: Beta = − 0.04, p = 0.03, serum albumin: Beta = 0.06, p = 0.07) (Fig. 1). There was no difference in the proportion of male to female offspring was identified in the control group (11F vs 10 M). However, a significant interaction with offspring gender in the GDM and T2DM groups (p = 0.02) was identified, with a higher proportion of female than male offspring in GDM participants (21F vs 11 M), whilst the opposite was true with T2DM participants (3F vs 11 M).

**Table 1:** General characteristics of participants

Time	Variables	Total	Controls	GDM	T2DM	F	P value	DM	F	P value
		(N = 67)	(N = 21)	(N = 32)	(N = 14)		ANOVA	(N = 46)		Controls vs DM
Second trimester	Age (years)	31.9 (5.7)	29.2 (5.1)	32.8 (5.6)	34.2 (5.5)	4.243	0.019	33.2 (5.6)	0.187	0.007
	BMI (kg/m2)	31.9 (6.8)	29.9 (8.4)	33.2 (6.1)	32.3 (5.0)	1.545	0.221	32.9 (5.7)	2.462	0.089
	SBP (mmHg)	111.6 (12.4)	109.9 (12.0)	109.7 (11.9)	118.5 (12.5)	2.913	0.062	112.3 (12.6)	0.017	0.459
	DBP (mmHg)	63.7 (7.7)	66.5 (9.3)	62.3 (5.7)	62.4 (8.2)	2.223	0.117	62.3 (6.5)	0.110	0.037
	Cholesterol (mmol.L)	4.8 (1.1)	5.0 (1.0)	4.7 (0.8)	4.9 (1.6)	0.449	0.783	4.7 (1.1)	4.220	0.552
	Triglycerides (mmol.L)	1.5 (0.8)	1.2 (0.6)	1.4 (0.9)	2.1 (0.5)	4.384	0.024	1.6 (0.8)	0.019	0.090
	HDL (mmol.L)	1.4 (0.4)	1.6 (0.4)	1.3 (0.3)	1.1 (0.5)	3.101	0.058	1.2 (0.4)	0.682	0.030
	LDL (mmol.L)	2.8 (0.9)	2.9 (0.9)	2.8 (0.7)	2.8 (1.4)	0.004	0.996	2.8 (1.0)	1.757	0.933
	Insulin (uIU.L)	0. 5 (0.4)	0.3 (0.3)	0.5 (0.3)	0.7 (0.5)	4.026	0.023	0.5 (0.4)	8.120	0.048
	HbA1C	5.4 (0.7)	5.1 (0.4)	5.3 (0.4)	6.0 (0.8)	11.976	0.000	5.5 (0.7)	2.943	0.02
	Vitamin D3	15.2 (6.0)	15.8 (5.6)	15.1 (6.8)	14.2 (4.3)	0.220	0.804	14.9 (6.2)	0.727	0.600
	Urea (nmol.L)	3.1 (1.2)	3.2 (1.0)	3.2 (1.5)	2.6 (0.4)	2.081	0.241	3.0 (1.2)	4.835	0.445
	Creatinine (nmol.L)	50.1 (9.1)	49.9 (8.1)	49.4 (10.1)	51.7 (8.9)	0.302	0.741	50.2 (9.7)	0.058	0.890
	ALT	15.2 (10.1)	13.2 (10.7)	17.0 (11.4)	14.6 (5.0)	0.894	0.414	16.2 (9.7)	0.609	0.269
	AST	16.8 (7.1)	16.3 (5.8)	18.1 (7.9)	14.9 (7.2)	1.010	0.370	17.0 (7.7)	0.248	0.700
	Bilirubin (umol.L)	7.6 (4.4)	7.8 (2.4)	7.9 (4.9)	6.8 (5.6)	0.305	0.721	7.5 (5.1)	2.879	0.813
	ALP (iu.L)	83.2 (35.5)	81.5 (41.7)	86.4 (34.9)	79.6 (27.7)	0.204	0.817	84.1 (32.4)	3.058	0.791
	Albumin (g.L)	34.7 (6.7)	36.5 (6.7)	35.0 (7.0)	31.7 (5.2)	2.251	0.114	33.9 (6.6)	2.328	0.145
	TSH (mU.L)	2.4 (3.4)	2.8 (3.5)	2.6 (4.0)	1.5 (0.7)	0.604	0.579	2.3 (3.4)	1.698	0.624
	Thyroxine (pmol.L)	12.6 (1.8)	12.9 (2.0)	12.4 (1.7)	12.6 (1.9)	0.283	0.666	12.4 (1.7)	0.065	0.388
	TP (g.L)	66.3 (5.4)	65.1 (5.2)	67.0 (5.7)	66.8 (4.8)	0.839	0.437	67.0 (5.4)	0.023	0.199
	Gestational age (weeks)	20.9 (4.4)	20.9 (4.9)	20.6 (4.3)	21.6 (3.9)	0.258	0.774	20.9 (4.2)	0.753	0.947
At birth	SBP (mmHg)	119.9 (11.8)	118.7 (11.8)	119.4 (9.5)	122.9 (16.4)	0.566	0.571	120.4 (11.8)	2.875	0.576
	DBP (mmHg)	72.1 (9.6)	70.4 (8.5)	72.4 (8.1)	74.1 (14.3)	0.618	0.542	72.9 (10.1)	6.333	0.328
	HbA1c	5.6 (0.9)	N/A	5.2 (0.4)	6.0 (1.3)	5.831	0.02	5.6 (0.9)	1.991	N/A
	Weight (Kg)	82.8 (14.0)	80.9 (17.0)	83.5 (12.7)	84.2 (12.6)	0.296	0.745	83.7 (12.6)	0.588	0.450
	Gestational age (weeks)	38.2 (1.6)	38.8 (1.2)	38.4 (1.4)	36.8 (1.6)	9.276	0.000	37.9 (1.6)	0.229	0.020
	Newborn weight (grams)	3073.9 (527.7)	3088.8 (492.2)	3110.3 (561.6)	2968.2 (523.1)	0.358	0.700	3067.0 (548.3)	0.356	0.877

BMI, body mass index; SBP, systolic blood pressure; DBP, diastolic blood pressure; LDL, low density lipoprotein; HDL, high density lipoprotein; HbA1c, glycated haemoglobin; TP, total protein; ALP, alkaline phosphatase; ALT, alanine transaminase; AST, aspartate aminotransferase. Data are presented as mean (SD). Differences between Controls, GDM and T2DM were tested by ANOVA. Differences between controls and all DM (GDM + T2DM) were tested by independent sample t test (normally distributed variables) or Mann–Whitney U (variables with skewed distribution) test. A p-value significance level of 0.05 was used

### A holistic view of the metabolic profile of GDM and T2DM

**Fig. 2:**
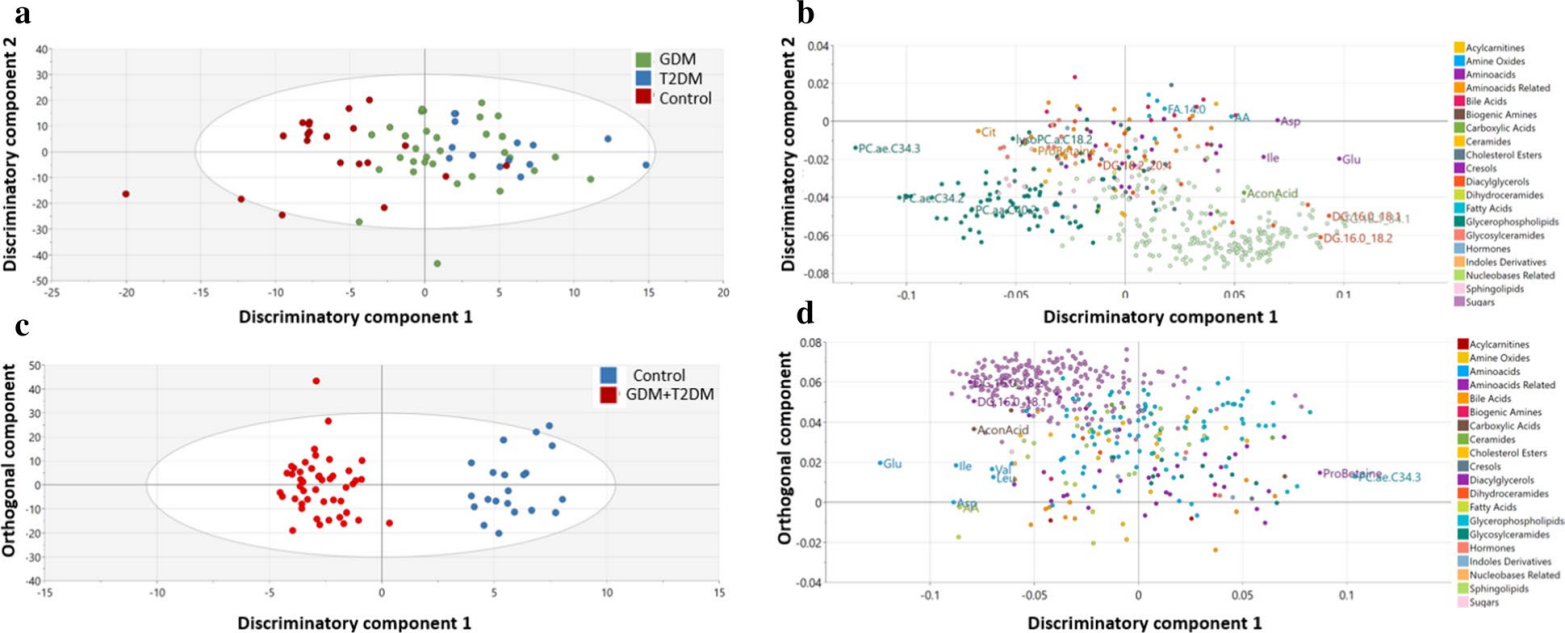
Orthogonal partial least square discriminate analysis (OPLS-DA) comparing metabolites from controls, GDM and T2DM individuals (top) and controls versus combined GDM + T2DM (bottom). Score plots from the two models respectively showing one dimensional separation of all three classes (control, GDM, T2DM) (**a**) and two classes (controls and GDM + T2DM) (**c**). The corresponding loading plots showing top associated metabolites differentiating controls, GDM and T2DM (**b**) or GDM + T2DM vs Controls (**d**) groups

OPLS-DA comparing the multivariate metabolic profile of controls, GDM and T2DM was used. OPLS-DA showed one class-discriminatory components accounting for 25% of the variation in the study group Y-variable. The Variable Importance in Projection (VIP) list emphasized the discriminatory effects of various glycerophospholipids (phosphatidylcholine acyl-alkyl, PC.ae) such as PC.ae.C34.3, PC.ae.C34.2 and PC.ae.C42.3, triacylglycerols (TG16.0/34.1, TG16.0/34.0 and TG18.1/32.0) and the amino acids, such as glutamate (Glu), aspartate (Asp), isoleucine (Ile) and arachidonic acid (AA) (Additional file 1: Table S1). The score plot in Fig. 2a indicates an x-axis that separates the controls from the GDM and T2DM. The corresponding loading plot, shown in Fig. 2b, indicates the aforementioned metabolites from the VIP list responsible for the groups’ separation. When GDM + T2DM (DM) were combined into one group, OPLS-DA showed one discriminatory component accounting for 92% of the variation in the control/combined DM group (Fig. 2c). The VIP list highlighted amino acids (Glu, leucine (Leu), valine (Val), Ile and Asp), triacylglycerols (TG16.0/34.2, TG16.0/34.1, TG18.2/32.0 and TG18.1/32.0) and the glycerophospholipid (C34:3) (Additional file 1: Table S2) [29], also indicated in the loading plot (Fig. 2d).

### Metabolites differentiating controls from GDM + T2DM

**Fig. 3:**
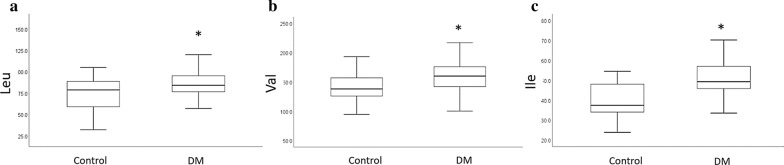
Boxplot of metabolites differentiating Control from DM (GDM + T2DM) groups. A Function enrichment analysis was performed using Fisher’s exact test by considering metabolites with a nominal p-value less than 0.1 from linear regression analysis. p-value significance level of 0.05 (*) was used

Following multivariate analysis, univariate regression models were used to characterize metabolite-level associations with GDM and DM (GDM + T2DM). The analysis revealed significant differences (false discovery rate (FDR) ≤ 0.05) in the levels of five metabolites between controls and T2DM (Additional file 1: Table S3). These include four amino acids (Asp, Glu, citrulline (Cit) and Ile) and the glycerophospholipid (PC.ae.C34.3). A number of metabolites differentiated controls from GDM, but none reached FDR level of significance (Additional file 1: Table S4). When comparing control vs DM, the linear model revealed a list of metabolites differentiating the two groups, with only Glu reaching FDR level of significance (FDR = 0.04) (Additional file 1: Table S5). Table 2 lists the top 10 metabolites differentiating control and DM with their sub and super-pathways. Enrichment analysis indicated that branched chain amino acids (BCAA) were elevated in DM compared to controls (p = 0.006) (Fig. 3).

**Table 2:** Top ten metabolites differentiating controls from DM (GDM + T2DM) in women in their second trimester

Metabolite	Biochemical ID	Super pathway	Sub pathway	Estimate	SE	P value	FDR
Glu	Glutamate	Amino Acids	Glutamate Metabolism	15.8	3.8	< 0.001	0.04
PC.ae.C34.3	Phosphatidylcholine C34:3	Lipid	Glycerophospholipids	− 1.5	0.4	< 0.001	0.13
Ile	Isoleucine	Amino Acids	Leucine, Isoleucine and Valine Metabolism	10.9	3.3	0.002	0.25
Asp	Aspartate	Amino Acids	Alanine and Aspartate Metabolism	1.3	0.5	0.005	0.29
Leu	Leucine	Amino Acids	Leucine, Isoleucine and Valine Metabolism	14.8	5.7	0.011	0.29
Val	Valine	Amino Acids	Leucine, Isoleucine and Valine Metabolism	23.5	8.9	0.011	0.29
ProBetaine	Proline betaine	Amino-Acid-related	Xenobiotic	− 2.5	1	0.011	0.29
AconAcid	Aconitate [cis or trans]	Carboxylic acids	TCA Cycle	0.3	0.1	0.009	0.29
DG.16.0/18.1	Palmitoyl-oleoyl-glycerol	Lipid	Diacylglycerol	1.7	0.6	0.009	0.29
DG.16.0/18.2	Palmitoyl-Linoleic acid-glycerol	Lipid	Diacylglycerol	1.1	0.4	0.006	0.29
AA	Arachidonate (20:4n6)	Fatty Acids	Long Chain Polyunsaturated Fatty Acid	0.4	0.1	0.003	0.29

Linear regression was performed to identify significant metabolites differentiating Controls from GDM + T2DM (DM) using the R statistical package after correcting for age, BMI and principle components (PC1 and PC2). Estimate (beta value), SE, standard error; FDR, False Discovery Rate

Furthermore, offspring gender interaction analysis revealed nominally significant association between metabolites and GDM + T2DM which were significantly different in pregnancies with male versus female offspring, including the triglycerides (18:2/31:0, 16:1/36:5, 17:1/36:4, 16:0/35:3, 18:2/33:0), the amino acids/related (glutamine and ProBetaine) and the cholesterol ester CE18:3 (Additional file 2: Figure S1).

### Metabolites associated with GA at delivery

**Fig. 4:**
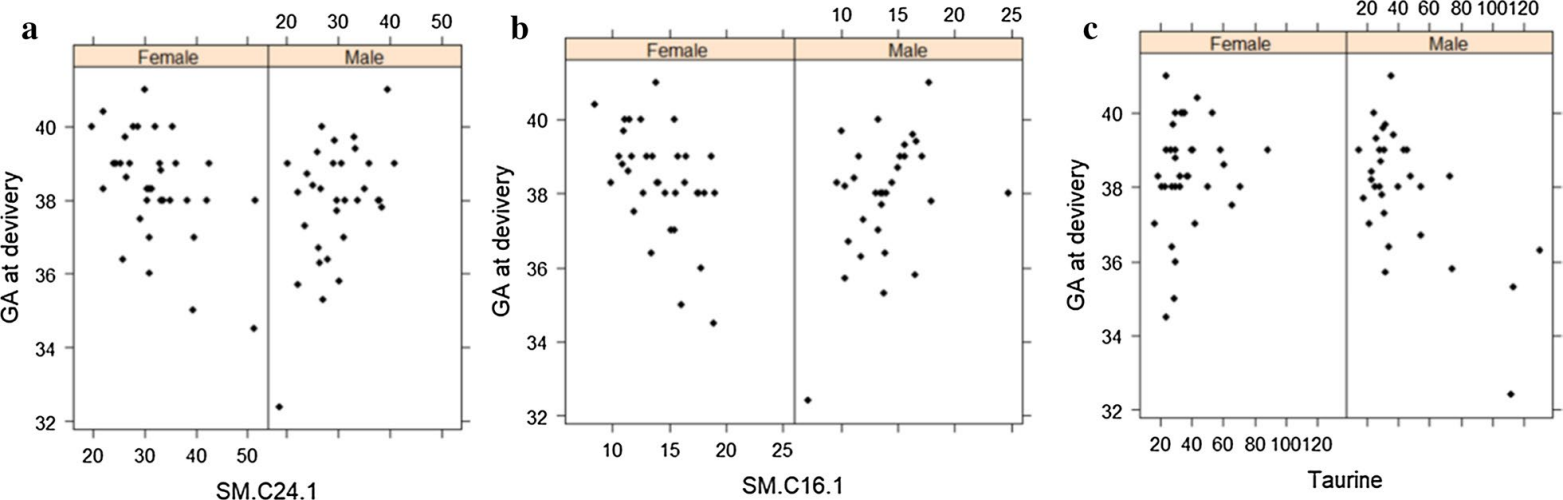
Gender specific associations with gestational age at delivery

Similarly, a linear model was used to assess the significance of metabolites associated with GA at delivery. One hundred and eleven metabolites exhibited significant association at FDR level of significance ≤ 0.05. The list of metabolites and their associated pathways are shown in Additional file 1: Table S6. Among these, 22 lipids were associated with gestational age at delivery at FDR ≤ 0.05 level of significance, including triacylglycerols, diacylglycerols and bile acids (Table 3). Among these, triacylglycerols and diacylglycerols containing C18:1 and C18:2 were enriched in pre-term deliveries (p < 0.01). Gender interaction analysis revealed three FDR significant metabolites where the slope of the regression line was significantly different between males and females in relation to GA at delivery. These include the sphingolipids (SM) C24:1 and C16:1 and the amino acid-related Taurine (Fig. 4). No significant associations were identified between metabolites and other adverse pregnancy outcomes, including pre-eclampsia, intrauterine fetal death, macrosomia, and maternal blood pressure at delivery (data not shown).

**Table 3:** Metabolites associated with gestational age at delivery

Metabolite	Biochemical ID	Super pathway	Sub pathway	Estimate	SE	P value	FDR
TG17.2/36.4	TG17:2/36:4	Lipid	Triaclylgerol	− 0.4	0.1	< 0.001	0.038
DG.18.1/18.1	DG18:1/18:1	Lipid	Diacylglycerol	− 1.7	0.5	0.002	0.044
DG.18.1/18.2	DG18:1/18:2	Lipid	Diacylglycerol	− 4.4	1.2	0.001	0.044
DG.18.2/18.2	DG18:2/18:2	Lipid	Diacylglycerol	− 2.8	0.9	0.002	0.044
TG16.0/36.3	TG16:0/36:3	Lipid	Triaclylgerol	− 157.6	43.0	0.001	0.044
TG16.1/36.3	TG16:1/36:3	Lipid	Triaclylgerol	− 12.8	3.8	0.001	0.044
TG18.0/36.3	TG18:0/36:3	Lipid	Triaclylgerol	− 7.9	2.3	0.001	0.044
TG18.1/34.1	TG18:1/34:1	Lipid	Triaclylgerol	− 194.1	57.2	0.001	0.044
TG18.1/34.2	TG18:1/34:2	Lipid	Triaclylgerol	− 132.1	39.4	0.001	0.044
TG18.1/36.1	TG18:1/36:1	Lipid	Triaclylgerol	− 14.0	4.3	0.002	0.044
TG18.1/36.2	TG18:1/36:2	Lipid	Triaclylgerol	− 53.1	15.5	0.001	0.044
TG18.1/36.3	TG18:1/36:3	Lipid	Triaclylgerol	− 61.7	16.2	< 0.001	0.044
TG18.2/34.1	TG18:2/34:1	Lipid	Triaclylgerol	− 125.3	36.6	0.001	0.044
TG18.2/36.0	TG18:2/36:0	Lipid	Triaclylgerol	− 1.0	0.3	0.002	0.044
TG18.2/36.1	TG18:2/36:1	Lipid	Triaclylgerol	− 9.7	3.0	0.002	0.044
TG18.2/36.2	TG18:2/36:2	Lipid	Triaclylgerol	− 31.2	9.6	0.002	0.044
TG18.2/38.4	TG18:2/38:4	Lipid	Triaclylgerol	− 1.9	0.5	0.001	0.044
TG20.0/32.4	TG20:0/32:4	Lipid	Triaclylgerol	− 0.5	0.1	0.002	0.044
TG20.4/36.2	TG20:4/36:2	Lipid	Triaclylgerol	− 3.7	1.1	0.001	0.044
GCA	Glycocholic acid	Lipid	Bile Acid	− 0.2	0.1	0.002	0.046
TG18.0/36.4	TG18:0/36:4	Lipid	Triaclylgerol	− 3.8	1.2	0.002	0.046

Linear regression was performed to identify significant metabolites associated with pre-term delivery using the R statistical package after correcting for age, BMI and principle components (PC1 and PC2). Estimate (beta value), SE, standard error; FDR, False Discovery Rate

### Predictive biomarkers association with GDM, DM and GA at delivery

**Fig. 5:**
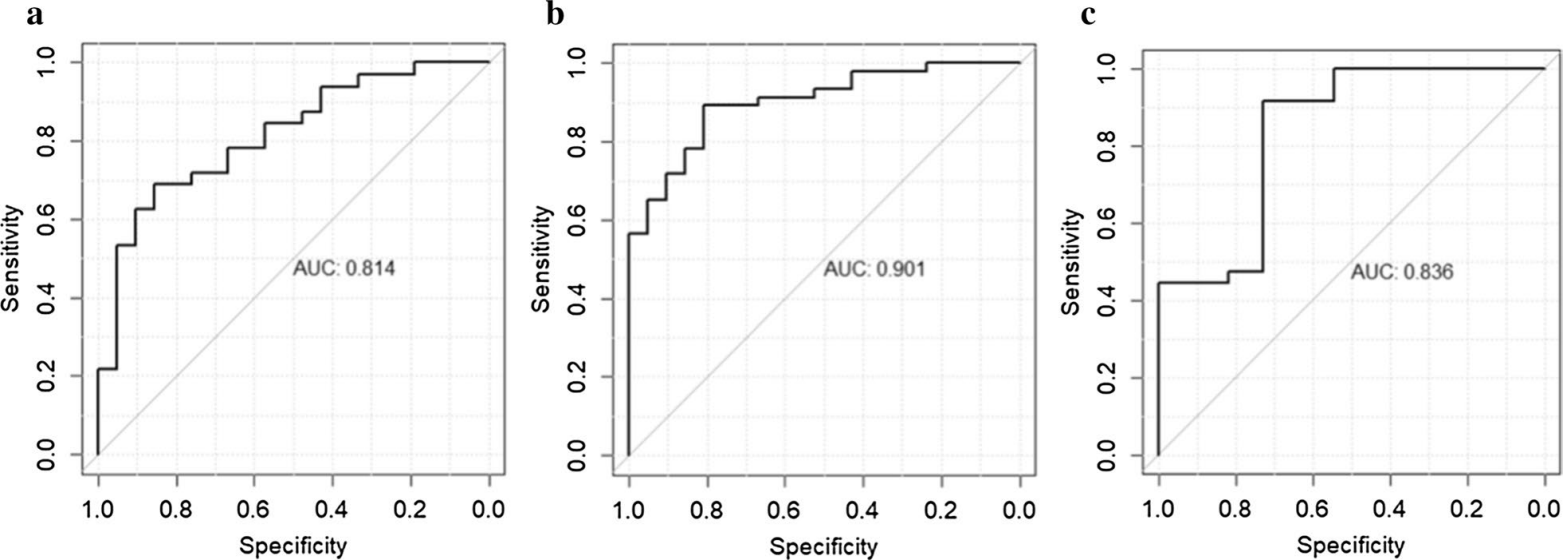
Predictors of GDM, DM and pre-term delivery. ROC curve indicates the best predictors of GDM (**a**), DM (**b**) and pre-term delivery (**c**) indicating the area under curve (AUC) for each model

We used an extension of the generalized linear model that features the elastic net regularization algorithm for variable selection, also known as GLMNET in R analysis software. The GLMNET model indicated that Glu (beta = 0.09, p = 0.001) was the best predictors of GDM compared to controls (AUC = 0.81) (Fig. 5a). The model also revealed that the phosphatidylcholine diacyl (PC.aa.C40.2) (beta = − 17.7, p = 0.05), arachidonic acid (AA) (beta = 3.2, pp = 0.003), glycochenodeoxycholic acid (GCDCA) (beta = 3.4, p = 0.02), and PC.ae.C34.3 (beta = 0.70 p = 0.02) were the best predictors of all DM compared to controls (AUC = 0.90) (Fig. 5b). The model also revealed that TG17.2/36.4 (beta = − 2.79, p0.03) and TG18.1/34.1 (beta = − 0.006, p = 0.01) were the best predictors of pre-term delivery (≤ 37 weeks) (AUC = 0.84) (Fig. 5c).

## Discussion

The diabetes epidemic constitutes a global public health challenge. Considering the adverse effects of DM on pregnancy outcomes, perinatal morbidity, and development of chronic diseases later in life, a better understanding of the metabolic mediators underlying these adverse effects could potentially provide novel diagnostic and therapeutic targets. In this study, targeted metabolomics of plasma samples from 67 pregnant women at second trimester was performed. Five metabolites exhibited significant differences between controls and T2DM, including four amino acids (Asp, Glu, Cit and Ile) and the glycerophospholipid (PC.ae.C34.3), whereas Glu was the only metabolite that significantly differentiated DM from controls. Glu was also found to be the best predictor of GDM by the GLMNET model confirmed by ROC analysis. The model also indicated that PC.aa.C40.2, AA, GCDCA, and PC.ae.C34.3 were the best predictors of all DM compared to controls. Our data also indicated that GA at delivery was on average 2 weeks earlier in T2DM than controls and GDM groups, which correlated negatively with HbA1c and SBP and positively with serum albumin at second trimester. GLMNET model confirmed by ROC analysis revealed that TG17.2/36.4 and TG18.1/34.1 were the best predictors of pre-term delivery (≤ 37 weeks). The potential functional relevance of identified metabolites in relation to diabetes and pre-term delivery is summarized in Table 4.

**Table 4:** The potential functional relevance of identified metabolites associated with diabetes and pre-term delivery

Metabolite	Association	Potential relevance to pathophysiological aspects of diabetes and pre-term delivery	References
Glu	Increased in DM	Activates N-methyl-D-aspartate receptors in β-cells, leading to acceleration of β-cell dysfunction and apoptosis induced by hyperglycemia	[36]
BCAA (valine, leucine, isoleucine)	Increased in DM	Promotes insulin resistance by modulating fatty acid oxidation, mTOR, JNK and IRS1 pathways	[39, 40]
Phosphatidylcholines	Decreased in DM	Serum antioxidants preventing lipoprotein oxidation	[41]
AA	Increased in DM	Arachidonic acid triggers insulin secretion, potentially increasing risk of insulin resistance	[42]
GCDCA	Increased in DM	Bile acids control gut bacteria overgrowth, species population, and protect the integrity of the intestinal barrier. Alterations in GCDCA can trigger diabetes	[43]
DG and TG containing C18:1 and C18:2	Increased in pre-term delivery	Serum linoleic acid is negatively correlated with visceral fat accumulation and risk of insulin resistance	[48]
TG17.2/36.4 and TG18.1/34.1	Best predictors of pre-term delivery	Remains to be investigated	

Glu, glutamate; BCAA, branched chain amino acids; mTOR, The mammalian target of rapamycin; JNK, c-Jun N-terminal kinase; IRS-1, insulin receptor substrate 1; AA, arachidonic acid; GCDCA, Glycochenodeoxycholic Acid; DG, diacylglycerols; TG, triacylglycerols

Metabolic signature of GDM, T2DM and DM (GDM + T2DM): In recent years, metabolomics has been widely used in the identification of novel pathways and specific biomarkers for insulin resistance and T2DM [22, 30–32]. The data presented here offers a holistic view of the changes in plasma metabolites in relation to GDM and T2DM in pregnant women in their second trimester compared to BMI and GA-matched controls. Our data revealed no FDR significant differences in metabolic profile between GDM and controls, perhaps due to the small sample size, although our data indicated that Glu was the best predictor of GDM in our study. When we combined GDM and T2DM into DM group, significant differences in Glu were observed between control and DM groups. The elevation in Glu in the DM group was previously reported in umbilical vein and artery as well as in the plasma of women with GDM compared to normal pregnant women [33, 34]. Glu is an important excitatory neurotransmitter with a potential role in diabetes development through excessive activation of N-methyl-D-aspartate receptors in β-cells and subsequent acceleration of β-cell dysfunction and apoptosis induced by hyperglycemia [35] (Table 4). Indeed, a previous report has indicated that elevated serum glu was associated with increased incidents of T2DM in 5 years follow up study [36]. Although measurement of a single amino acid is unlikely to be sufficient to differentiate controls from patients, future studies validating the potential use of glu as well as other associated metabolites as predictors of GDM before onset and the impact of targeting glutamate to reduce risk of T2DM are warranted. Our data also indicated an enrichment of the BCAAs (valine, leucine and isoleucine) in DM compared to controls. Previous epidemiological studies have indicated that elevated levels of circulating BCAAs are associated with insulin resistance and T2DM, perhaps because of altered energy metabolism or dietary habits [37, 38]. BCAAs were shown previously to be involved in several pathways of insulin resistance, including fatty acid oxidation, mTOR, JNK and IRS1 pathways [39, 40] (Table 4). The phosphatidylcholines PC.aa.C40.2 and PC.ae.C34.3 as well as AA and GCDCA were identified as the best predictors of all DM compared to controls. Similar to our findings, previous studies have indicated an inverse relationship between acyl-alkyl-phosphatidylcholines C34:3, C40:6, C42:5, C44:4, and C44:5 and T2DM risk [35]. Phosphatidylcholines are a major constituent of cell membranes. They play an important role in membrane-mediated cell signaling and Phosphatidylcholine Transfer Protein activation of other enzymes. Their decrease in T2DM could be due to their role as serum antioxidants preventing lipoprotein oxidation [41] (Table 4). Additionally, elevated arachidonic acid levels during glucose-induced insulin release were previously shown to trigger further increases in insulin secretion, potentially increasing risk of insulin resistance [42] (Table 4). This could explain why AA was identified as one of the top predictors of DM in our study. Alterations in GCDCA, amongst other bile acids, was previously shown to trigger diabetes [43], which could also explain why it appeared as one of the top predictors of DM in our study (Table 4). When

### Determinants of pre-term delivery

GA at delivery was on average 2 weeks earlier in T2DM than controls and GDM groups. It negatively correlated with HbA1c at second trimester, confirming previous findings of inverse correlation between HbA1C concentration and length of gestation from early pregnancy to mid-3rd trimester [44]. GA at delivery also correlated negatively with SBP at second trimester, which also confirmed previous reports of an association between elevations in SBP in 3rd trimester with spontaneous pre-term births [45]. Interestingly, a significant inverse correlation between SBP and gestational age at birth has been consistently observed from childhood to adulthood in pre-term-born individuals [46]. Our data also showed a positive correlation between serum albumin and pre-term labor. This observation also confirms previous data suggesting that woman with higher serum albumin levels at the second visit had a longer pregnancy duration, possibly reflecting better nutritional status [47]. Twenty-two metabolites were significantly associated with pre-term delivery, including triacylglycerols and diacylglycerols containing C18:1 (oleic acid) and C18:2 (linoleic acid). Our data agree with previous studies suggesting a negative correlation between linoleic acid levels and reduction of insulin resistance [48] (Table 4). The GLMNET model revealed that TG17.2/36.4 and TG18.1/34.1 are the best predictors of pre-term delivery (≤ 37 weeks). Whether these metabolic differences were due to T2DM or just gestation age remains to be investigated, as both gestational age and T2DM will strongly influence the dynamics of metabolites in pregnant women. Further quantitative studies will be required to determine if the detection of these compounds may be a valuable clinical predictor of premature delivery in the second trimester, or earlier.

Offspring gender interacting metabolites: Our data indicated a significant interaction with offspring gender in women with GDM and T2DM as higher proportion of female than male offspring were identified in GDM participants, but more males than females in T2DM participants. Previous studies have found that women carrying male fetuses were more likely to have gestational diabetes [49, 50]. The results from these studies agree with our data from T2DM women but not GDM counterparts, however as the numbers of participating women in each group are small, a confirmation in a larger cohort is warranted. When considering metabolites that exhibit significant association with gender in GDM and T2DM women, a number of metabolic differences were identified in pregnancies with male versus female offspring, including specific triglycerides, amino acids and the cholesterol esters. When considering metabolites that show gender interaction with GA at delivery, three metabolites were identified. These included the sphingolipids (SM) C24:1 and C16:1 that exhibited significant opposite direction of correlation between males and females, and the amino acid-related Taurine that was only significantly negatively correlated with GA at delivery in males. The functional relevance of these interactions remain to be investigated.

### Study limitations

The relatively low number of participants per group was a main limitation of our study, which was potentially responsible for lack of detected differences between GDM and the control group; however, multiple significant associations were identified between metabolites and pre-term delivery. In order to enhance the power to identify significant differences between controls and DM, GDM and T2DM were combined into one group since GDM is associated with both insulin resistance and impaired insulin secretion and shares the same risk factors with T2DM [51]. Additionally, the cross-sectional nature of the study limited the assessment of the evolutionary process of metabolites throughout pregnancy and the interpretation of the findings from a pathophysiological point of view. The observational nature of the findings dictates functional validation before suggesting any causalities. Furthermore, since blood samples were collected at multiple sites, a batch effect may have occurred, but this was mitigated by standardized protocols for sample collection, processing and storage. It is possible that other unmeasured factors may have influenced our data including dietary habits, medication/supplements and other unknown environmental factors; however, inclusion of principle components in the regression model may have captured part of these potential confounding factors. Finally, due to the limited sample size, splitting the cohort into testing and validation was not possible, therefore the ROC curve analysis was used on the full dataset to examine the discriminatory ability of metabolites that were detected as significant from regression analysis based on the same data. A more rigorous validation of the results is warranted and requires a separate cohort. Large cohorts, dynamic monitoring of metabolites during pregnancy, and analyses of various specimen types could improve our understanding of metabolites alteration and verify the validity of multi-marker predictive models of GDM and pre-term labor. Such dynamic monitoring would also enable further mitigation of the impact on these metabolic changes on both mothers and their fetuses. Furthermore, comparing short and long-term post-delivery effects would provide additional support for measurement of critical biomarkers and development of guidelines and methods to mitigate these effects.

## Conclusion

Our data provided a comprehensive overview of metabolite alteration in women in their second trimester, with metabolic profiling identifying significant associations between a number of metabolites and T2DM/GDM patients including glutamate, branched chair amino acids, phosphatidylcholines and certain triglycerides. Future studies are warranted to confirm and validate these markers in large cohorts and different ethnicities and to study their potential utilization for targeted therapies.

## Supplementary information

**Additional file 1: Table S1.** Variable Importance in Projection (VIP) list from OPLS-DA loading plot for controls vs GDM vs T2DM. **Table S2.** Variable Importance in Projection (VIP) list from OPLS-DA loading plot for controls vs DM (GDM+T2DM). **Table S3.** Metabolites differentiating controls vs T2DM. **Table S4.** Metabolites differentiating controls vs GDM. **Table S5.** Metabolites differentiating controls vs DM (GDM+T2DM). **Table S6.** Metabolites associated with GA at delivery.

**Additional file 2: Figure S1.** Gender specific associations with combined GDM+T2DM groups. The metabolites shown scored a nominal anova pvalue < 0.01 from the interaction term (gender:group) and show differential pattern of associations with diabetes status per gender group. The ANOVA p values for interaction effects is TG.18.2_31.0 (0.001199), ProBetaine (0.0032), CE.18.3 (0.004137), TG.16.1_36.5(0.005635), Gln (0.005895),TG.17.1_36.4(0.005918), TG.16.0_35.3 (0.0071),TG.18.2_33.0 (0.0092). The * denotes the significant contrasts for each gender group.

## Data Availability

The datasets used and/or analysed during the current study are available from the corresponding author on reasonable request.

## Abbreviations

AconAci: Aconitate
ALT: Alanine transaminase
ALP: Alkaline phosphatase
AA: Arachidonic acid
AUC: Area under curve
Asp: Aspartate
AST: Aspartate aminotransferase
BMI: Body mass index
BCAA: Branched chain amino acids
CVD: Cardiovascular disease
Cit: Citrulline
DM: Diabetes Mellitus
DG: Diacylglycerol
DBP: Diastolic blood pressure
ESI: Electrospray ionization
FDR: False discovery rate
FBG: Fasting blood glucose
FIA-MS/MS: Flow Injection Analysis Tandem Mass Spectrometry
GA: Gestational age
GDM: Gestational diabetes mellitus
Glu: Glutamate
HbA1c: Glycated haemoglobin
GCDCA: Glycochenodeoxycholic acid
GCA: Glycocholic acid
GCP: Good Clinical Practice
HDL: High density lipoprotein
IFG: Impaired fasting glucose
IGT: Impaired glucose tolerance
ITEM: Institute for Toxicology and Experimental Medicine
ICH: International Council for Harmonisation
Ile: Isoleucine
Leu: Leucine
LDL: Low density lipoprotein
MS: Mass spectrometry
MRM: Multiple reaction monitoring
OGTT: Oral glucose tolerance test
OPLS-DA: Orthogonal partial least square discriminant analysis
PC.ae: Phosphatidylcholine acyl-alkyl
PC.aa: Phosphatidylcholine diacyl
PCA: Principle component analysis
ROC: Receiver operating characteristic curve
SBP: Systolic blood pressure
TP: Total protein
TG: Triacylglycerols
T2DM: Type 2 diabetes mellitus
Val: Valine
VIP: Variable Importance in Projection
WHO: World health organization

## References

[1] American Diabetes A (2003). Gestational diabetes mellitus. Diabetes Care.

[2] Abdi H, Hosseinpanah F, Azizi F, Hadaegh F, Amouzegar A (2017). Screening for dysglycemia: a comment on classification and diagnosis of diabetes in american diabetes association standards of medical care in diabetes-2016. Arch Iran Med.

[3] Zhu Y, Zhang C (2016). Prevalence of gestational diabetes and risk of progression to type 2 diabetes: a global perspective. Curr Diab Rep.

[4] Bashir M, Abdel-Rahman EM, Aboulfotouh M, Eltaher F, Omar K, Babarinsa I, Appiah-Sakyi K, Sharaf T, Azzam E, Abukhalil M, Boumedjane M (2018). Prevalence of newly detected diabetes in pregnancy in Qatar, using universal screening. PLoS ONE.

[5] ACOG Practice Bulletin No. 190 (2018). Gestational diabetes mellitus. Obstet Gynecol.

[6] Pallardo F, Herranz L, Garcia-Ingelmo T, Grande C, Martin-Vaquero P, Janez M, Gonzalez A (1999). Early postpartum metabolic assessment in women with prior gestational diabetes. Diabetes Care.

[7] Patti AM, Pafili K, Papanas N, Rizzo M (2018). Metabolic disorders during pregnancy and postpartum cardiometabolic risk. Endocr Connect.

[8] Garcia-Vargas L, Addison SS, Nistala R, Kurukulasuriya D, Sowers JR (2012). Gestational diabetes and the offspring: implications in the development of the cardiorenal metabolic syndrome in offspring. Cardiorenal Med.

[9] Hedderson MM, Ferrara A, Sacks DA (2003). Gestational diabetes mellitus and lesser degrees of pregnancy hyperglycemia: association with increased risk of spontaneous preterm birth. Obstet Gynecol.

[10] Yogev Y, Langer O (2007). Spontaneous preterm delivery and gestational diabetes: the impact of glycemic control. Arch Gynecol Obstet.

[11] Lepercq J, Coste J, Theau A, Dubois-Laforgue D, Timsit J (2004). Factors associated with preterm delivery in women with type 1 diabetes: a cohort study. Diabetes Care.

[12] Kock K, Kock F, Klein K, Bancher-Todesca D, Helmer H (2010). Diabetes mellitus and the risk of preterm birth with regard to the risk of spontaneous preterm birth. J Matern Fetal Neonatal Med.

[13] Al-Wassia H, Saber M (2017). Admission of term infants to the neonatal intensive care unit in a Saudi tertiary teaching hospital: cumulative incidence and risk factors. Ann Saudi Med.

[14] Huang YT, Lin HY, Wang CH, Su BH, Lin CC (2018). Association of preterm birth and small for gestational age with metabolic outcomes in children and adolescents: a population-based cohort study from Taiwan. Pediatr Neonatol.

[15] Louise T, Nauf Bendar AS, Durighel G, Frost G, Bell J (2012). The effect of preterm birth on adiposity and metabolic pathways and the implications for later life. Clin Lipidol.

[16] Crump C, Sundquist J, Howell EA, McLaughlin MA, Stroustrup A, Sundquist K (2020). Pre-term delivery and risk of ischemic heart disease in women. J Am Coll Cardiol.

[17] Berger H, Melamed N (2014). Timing of delivery in women with diabetes in pregnancy. Obstet Med.

[18] Feghali MN, Caritis SN, Catov JM, Scifres CM (2016). Timing of delivery and pregnancy outcomes in women with gestational diabetes. Am J Obstet Gynecol.

[19] Solomon CG, Willett WC, Carey VJ, Rich-Edwards J, Hunter DJ, Colditz GA, Stampfer MJ, Speizer FE, Spiegelman D, Manson JE (1997). A prospective study of pregravid determinants of gestational diabetes mellitus. JAMA.

[20] Guasch-Ferre M, Hruby A, Toledo E, Clish CB, Martinez-Gonzalez MA, Salas-Salvado J, Hu FB (2016). Metabolomics in prediabetes and diabetes: a systematic review and meta-analysis. Diabetes Care.

[21] Al-Sulaiti H, Diboun I, Banu S, Al-Emadi M, Amani P, Harvey TM, Domling AS, Latiff A, Elrayess MA (2018). Triglyceride profiling in adipose tissues from obese insulin sensitive, insulin resistant and type 2 diabetes mellitus individuals. J Transl Med.

[22] Al-Sulaiti H, Diboun I, Agha MV, Mohamed FFS, Atkin S, Domling AS, Elrayess MA, Mazloum NA (2019). Metabolic signature of obesity-associated insulin resistance and type 2 diabetes. J Transl Med.

[23] Plows JF, Stanley JL, Baker PN, Reynolds CM, Vickers MH (2018). The pathophysiology of gestational diabetes mellitus. Int J Mol Sci..

[24] Huynh J, Xiong G, Bentley-Lewis R (2014). A systematic review of metabolite profiling in gestational diabetes mellitus. Diabetologia.

[25] Mierzyński R, Dłuski D, Nowakowski Ł, Poniedziałek-Czajkowska E, Leszczyńska-Gorzelak B (2018). Adiponectin and omentin levels as predictive biomarkers of preterm birth in patients with gestational diabetes mellitus. Biomed Res Int.

[26] Mahajan UV, Varma VR, Huang CW, An Y, Tanaka T, Ferrucci L, Takebayashi T, Harada S, Iida M, Legido-Quigley C, Thambisetty M (2020). Blood metabolite signatures of metabolic syndrome in two cross-cultural older adult cohorts. Int J Mol Sci..

[27] Feng C, Wang H, Lu N, Chen T, He H, Lu Y, Tu XM (2014). Log-transformation and its implications for data analysis. Shanghai Arch Psychiatry.

[28] Wishart DS, Tzur D, Knox C, Eisner R, Guo AC, Young N, Cheng D, Jewell K, Arndt D, Sawhney S (2007). HMDB: the human metabolome database. Nucleic Acids Res.

[29] Mohamed E, Ilhame D, Manjunath R, Yasser M, Lina A, Mohammed B, Alexandra EB, Abdul-Badi A-S, Stephen LA, Nayef AM, Mohamed AE. Metabolic profiling of pre-gestational and gestational diabetes mellitus identifies novel predictors of pre-term labor: Supplementary tables S1–S5. 2020.

[30] Halama A, Kulinski M, Kader SA, Satheesh NJ, Abou-Samra AB, Suhre K, Mohammad RM (2016). Measurement of 1,5-anhydroglucitol in blood and saliva: from non-targeted metabolomics to biochemical assay. J Transl Med.

[31] Wang-Sattler R, Yu Z, Herder C, Messias AC, Floegel A, He Y, Heim K, Campillos M, Holzapfel C, Thorand B (2012). Novel biomarkers for pre-diabetes identified by metabolomics. Mol Syst Biol.

[32] Arneth B, Arneth R, Shams M (2019). Metabolomics of type 1 and type 2 diabetes. Int J Mol Sci.

[33] Cetin I, de Santis MS, Taricco E, Radaelli T, Teng C, Ronzoni S, Spada E, Milani S, Pardi G (2005). Maternal and fetal amino acid concentrations in normal pregnancies and in pregnancies with gestational diabetes mellitus. Am J Obstet Gynecol.

[34] Rahimi N, Razi F, Nasli-Esfahani E, Qorbani M, Shirzad N, Larijani B (2017). Amino acid profiling in the gestational diabetes mellitus. J Diabetes Metab Disord.

[35] Huang XT, Li C, Peng XP, Guo J, Yue SJ, Liu W, Zhao FY, Han JZ, Huang YH, Yang L (2017). An excessive increase in glutamate contributes to glucose-toxicity in beta-cells via activation of pancreatic NMDA receptors in rodent diabetes. Sci Rep.

[36] Chen S, Akter S, Kuwahara K, Matsushita Y, Nakagawa T, Konishi M, Honda T, Yamamoto S, Hayashi T, Noda M, Mizoue T (2019). Serum amino acid profiles and risk of type 2 diabetes among Japanese adults in the Hitachi Health Study. Sci Rep.

[37] Karusheva Y, Koessler T, Strassburger K, Markgraf D, Mastrototaro L, Jelenik T, Simon MC, Pesta D, Zaharia OP, Bodis K (2019). Short-term dietary reduction of branched-chain amino acids reduces meal-induced insulin secretion and modifies microbiome composition in type 2 diabetes: a randomized controlled crossover trial. Am J Clin Nutr.

[38] Flores-Guerrero JL, Osté MC, Kieneker LM, Gruppen EG, Wolak-Dinsmore J, Otvos JD, Connelly MA, Bakker SJ, Dullaart RP (2018). Plasma branched-chain amino acids and risk of incident type 2 diabetes: results from the PREVEND prospective cohort study. J Clin Med..

[39] Copps KD, White MF (2012). Regulation of insulin sensitivity by serine/threonine phosphorylation of insulin receptor substrate proteins IRS1 and IRS2. Diabetologia.

[40] Newgard CB (2012). Interplay between lipids and branched-chain amino acids in development of insulin resistance. Cell Metab.

[41] Wallner S, Schmitz G (2011). Plasmalogens the neglected regulatory and scavenging lipid species. Chem Phys Lipids.

[42] Persaud SJ, Muller D, Belin VD, Kitsou-Mylona I, Asare-Anane H, Papadimitriou A, Burns CJ, Huang GC, Amiel SA, Jones PM (2007). The role of arachidonic acid and its metabolites in insulin secretion from human islets of langerhans. Diabetes.

[43] Ferrell JM, Chiang JYL (2019). Understanding bile acid signaling in diabetes: from pathophysiology to therapeutic targets. Diabetes Metab J.

[44] Arbib N, Shmueli A, Salman L, Krispin E, Toledano Y, Hadar E (2019). First trimester glycosylated hemoglobin as a predictor of gestational diabetes mellitus. Int J Gynaecol Obstet.

[45] Zhang J, Villar J, Sun W, Merialdi M, Abdel-Aleem H, Mathai M, Ali M, Yu KF, Zavaleta N, Purwar M (2007). Blood pressure dynamics during pregnancy and spontaneous preterm birth. Am J Obstet Gynecol.

[46] Sutherland MR, Bertagnolli M, Lukaszewski MA, Huyard F, Yzydorczyk C, Luu TM, Nuyt AM (2014). Preterm birth and hypertension risk: the oxidative stress paradigm. Hypertension.

[47] Forest JC, Masse J, Moutquin JM (1996). Maternal hematocrit and albumin as predictors of intrauterine growth retardation and preterm delivery. Clin Biochem.

[48] Fujii C, Kawai T, Azuma K, Oguma Y, Katsukawa F, Hirose H, Tanaka K, Meguro S, Matsumoto H, Itoh H (2017). Relationships between composition of major fatty acids and fat distribution and insulin resistance in Japanese. J Diabetes Res.

[49] Sheiner E, Levy A, Katz M, Hershkovitz R, Leron E, Mazor M (2004). Gender does matter in perinatal medicine. Fetal Diagn Ther.

[50] Ehrlich SF, Eskenazi B, Hedderson MM, Ferrara A (2012). Sex ratio variations among the offspring of women with diabetes in pregnancy. Diabet Med.

[51] Herath H, Herath R, Wickremasinghe R (2017). Gestational diabetes mellitus and risk of type 2 diabetes 10 years after the index pregnancy in Sri Lankan women-A community based retrospective cohort study. PLoS ONE.

