# Supplementary figures and images for: Metabolic profiling of pre-gestational and gestational diabetes mellitus identifies novel predictors of pre-term delivery
Source: J Transl Med. 2020 Sep 24;18:366. doi: 10.1186/s12967-020-02531-5 (PMC7517617; doi:10.1186/s12967-020-02531-5)

Figure S1.


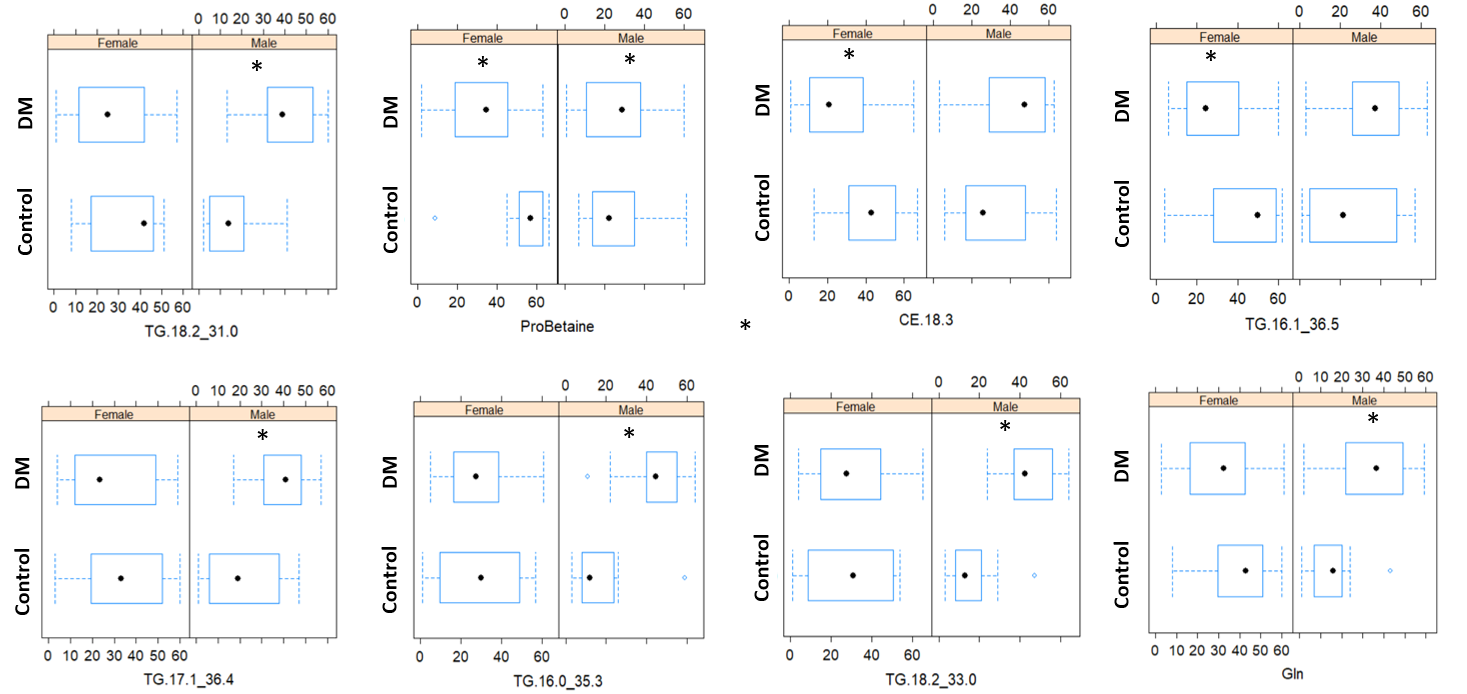

Supplement: Supplementary file 2 — Additional file 2: Figure S1. Gender specific associations with combined GDM+T2DM groups. The metabolites shown scored a nominal anova pvalue < 0.01 from the interaction term (gender:group) and show differential pattern of associations with diabetes status per gender group. The ANOVA p values for interaction effects is TG.18.2_31.0 (0.001199), ProBetaine (0.0032), CE.18.3 (0.004137), TG.16.1_36.5(0.005635), Gln (0.005895),TG.17.1_36.4(0.005918), TG.16.0_35.3 (0.0071),TG.18.2_33.0 (0.0092). The * denotes the significant contrasts for each gender group. [file 12967_2020_2531_MOESM2_ESM.docx]
